# Molecular comparison of pure ovarian fibroma with serous benign ovarian tumours

**DOI:** 10.1186/s13104-020-05194-z

**Published:** 2020-07-22

**Authors:** Sally M. Hunter, Genevieve V. Dall, Maria A. Doyle, Richard Lupat, Jason Li, Prue Allan, Simone M. Rowley, David Bowtell, On behalf of AOCS, Ian G. Campbell, Kylie L. Gorringe

**Affiliations:** 1 Cancer Genomics Program, Peter MacCallum Cancer Centre, East Melbourne, Australia; 2 Bioinformatics Core Facility Peter MacCallum Cancer Centre, East Melbourne, Victoria, Australia; 3 Anatomical Pathology, Peter MacCallum Cancer Centre, East Melbourne, Victoria, Australia; 4 The Department of Pathology, University of Melbourne, Parkville, Australia; 5 The Sir Peter MacCallum Department of Oncology, University of Melbourne, Parkville, Australia; 6 Peter MacCallum Cancer Centre, Locked Bag 1, A’Beckett Street, Melbourne, VIC 8006 Australia

**Keywords:** Ovarian fibroma, Adenofibroma, Cystadenomas, Cystadenofibroma, Copy number aberrations, Gene expression, Exome sequencing, Microarrays

## Abstract

**Objective:**

Ovarian fibromas and adenofibromas are rare ovarian tumours. They are benign tumours composed of spindle-like stromal cells (pure fibroma) or a mixture of fibroblast and epithelial components (adenofibroma). We have previously shown that 40% of benign serous ovarian tumours are likely primary fibromas due to the neoplastic alterations being restricted to the stromal compartment of these tumours. We further explore this finding by comparing benign serous tumours to pure fibromas.

**Results:**

Performing copy number aberration (CNA) analysis on the stromal component of 45 benign serous tumours and 8 pure fibromas, we have again shown that trisomy of chromosome 12 is the most common aberration in ovarian fibromas. CNAs were more frequent in the pure fibromas than the benign serous tumours (88% vs 33%), however pure fibromas more frequently harboured more than one CNA event compared with benign serous tumours. As these extra CNA events observed in the pure fibromas were unique to this subset our data indicates a unique tumour evolution. Gene expression analysis on the two cohorts was unable to show gene expression changes that differed based on tumour subtype. Exome analysis did not reveal any recurrently mutated genes.

## Introduction

Ovarian fibromas and adenofibromas form part of the sex-cord stromal family of tumours and are relatively uncommon, accounting for approximately 8% of all diagnosed ovarian tumours [1]. These tumours are benign entities composed in significant part of fibroblasts (pure fibromas), or as compound tumours composed of a mix of fibroblast and epithelial (adenofibroma) or sex-cord (granulosa-stromal tumours) components. Tumours with a cystic epithelial component are termed cystadenomas or cystadenofibromas.

Due to their relative rarity and benign nature these tumours have not been well molecularly characterised, with the majority of studies focussing on immunohistochemistry and cytogenetics. Ovarian fibromas differ from fibromas arising in other organs in that they frequently express hormone receptors (e.g. ER-β, PR, AR) and are typically negative for the characteristic markers of other cells derived from a fibroblast/myofibroblastic origin (e.g. SMA, CD34, CD117, S-100) [1, 2].

Genomic aberrations involving trisomy and tetrasomy of chromosome 12 appear to be particularly prevalent in tumours arising in the female genitourinary tract, including uterine leiomyomas [3, 4], thecomas [5–7], fibromas [7–10] and granulosa cell tumours [11, 12]. The underlying biological driver for this recurrent event has yet to be established. Other genomic aberrations that arise less frequently may be more cell type-specific. Imbalances involving chromosomes 4 and 9 are also common in the fibroma-thecoma subgroup, with chromosome 9 aberrations potentially being associated with cellular fibromas [13].

We previously reported that around 40% of benign serous cystadenomas and cystadenofibromas show copy number aberrations (CNA) exclusively in the stroma [14] and are thus likely misdiagnosed primary fibromas with epithelial inclusion. To investigate this further we undertook molecular characterisation of these epithelial-stromal tumours in comparison to pure fibromas.

## Main text

### Materials and methods

#### Tissue samples

Fresh frozen tissue samples were used for copy number, exome and expression analyses. All samples were collected with the patient’s informed consent and the study was approved by the Human Research Ethics Committees at the Peter MacCallum Cancer Centre. Patients with ovarian tumors were identified through hospitals in the Wessex Region, UK (n = 25) [15] and the Australian Ovarian Cancer Study (AOCS) (n = 31) [16, 17]. Pathology review was conducted on cryosections adjacent to the tissue from which DNA was extracted (PA). Microdissections and DNA/RNA extractions were performed as previously described [18]. Samples were selected for inclusion based on availability of tissue for DNA and RNA extraction.

#### Copy number data

The Affymetrix SNP6.0 Human Mapping (1.8 M probe set) array was utilised for ultra-high resolution allele-specific copy number analysis. Arrays were performed as recommended by the manufacturer with the exception that the input was reduced from the recommended 500 ng to 250 ng by reducing reaction volumes by half for all processes prior to the SNP6.0 PCR step. Reduction in DNA input does not result in any loss in the quality of the data. Copy number analysis was performed as previously described [18], using Partek Genomics Suite v 6.5. Copy number and allele-specific copy number was generated paired (when matching normal available) or unpaired and circular binary segmentation was performed to identify regions of copy number and loss of heterozygosity. Thresholds were > 2.3 for gains, < 1.7 for losses and < 0.75 for homozygous deletions. All SNP data has been made publicly available through Gene Expression Omnibus (http://www.ncbi.nlm.nih.gov/geo/ - GSE67189).

#### Exome sequencing

For each case 500 ng–1 µg of microdissected tumour DNA and matched lymphocyte DNA when available was sheared to < 1000 bp using a Covaris^®^ ultra-sonicator (Covaris^®^), libraries prepared using the Illumina TruSeq DNA Sample Preparation procedure (Illumina), and enriched for exome sequencing using the SeqCap EZ Human Exome Library v2.0 (Roche NimbleGen). Exomes were sequenced with 100 bp PE reads in pools of three per lane on a HiSeq 2000 (Illumina).

Sequence reads were aligned to the human genome (GRCh37/hg19) using BWA-MEM (v0.7.7-r441) [19, 20]; duplicates marked using Picard (v1.77); local indel realignment and base quality recalibration performed using GATK (v2.7-2-g6bda569) [21, 22]; indel detection performed using GATK Unified Genotyper (v2.7-2-g6bda569), Indel Genotyper, Pindel (v0.2.5a3) [23], and VarScan2 (v2.2.4) [24]; SNV prediction performed using GATK Unified Genotyper, MuTect (v2.7-1-g42d771f) [25], SomaticSniper [26], JointSNVMix2 (v0.8-b2) [27], and VarScan2 (v2.2.4); and variants annotated using Ensembl variant effect predictor v73. Exome bam files are available from the Sequence Read Archive Accession number PRJNA631561 (https://www.ncbi.nlm.nih.gov/sra/PRJNA631561).

Variants were enriched for genuine somatic events by filtering for those called by >=2 variant callers, with the exception of MuTect, which is capable of detecting variants at lower frequencies and therefore all MuTect variants were included; germline allele frequency <=0.01 and tumour allele frequency >=0.05, with >=0.1 difference in allele frequency between tumour and germline; variant observed in <=3 of 250 in-house germ-line exomes. All variants with a tumour allele frequency >=0.1 were taken forward for Sanger sequencing validation.

#### Expression data

Expression data was generated using the Affymetrix Human Gene 1.0 ST array according to the manufacturer’s recommendations. An input of 300 ng of total RNA was used, as quantified by Nanodrop spectrophotometer. RIN values were determined using the Agilent Bioanalyzer RNA 6000 Nano assay, the average RIN value for the 25 samples was 4.7 (range 1–7.9). Analysis of the data was performed using the Partek Gene Expression workflow. CEL files were processed using RMA normalisation and batch correction. All Gene 1.0 ST data has been made publicly available through Gene Expression Omnibus (http://www.ncbi.nlm.nih.gov/geo/-GSE67223).

### Results

The clinical features of the ovarian cohort are presented in Table 1. Women with ovarian fibromas compared to benign serous ovarian tumours had very similar median ages and ranges (64, range 35–80 and 61, range 27–80 respectively). Interestingly, there appeared to be a strong preponderance for both fibromas and benign serous tumours to be bilateral or detected on the right ovary (Binomial test for left vs right P = 0.02).

**Table 1:** Clinical features of cohort

Sample ID	Age	Laterality	CN	Gene expression	Exome
Pure Fibromas					
IC33	80	Bilateral (same)	Y	Y	
IC4	n/a	Bilateral (same)	Y	Y	
IC269	59	Right	Y	Y	
IC425	49	Left	Y	Y	
IC458	35	Right	Y	Y	
IC494	53	Bilateral (same)	Y	Y	
IC181	65	Right	Y	Y	
IC137	64	Bilateral (other)	Y	Y	
Cystadenofibroma					
IC149	66	Bilateral (same)	Y	Y	
IC10	59	Right	Y	Y	
IC164	72	Left	Y^a^	Y	Y
IC158	82	Bilateral (same)	Y^a^	Y	Y
IC5	81	Left	Y^a^	Y	Y
IC103	56	Bilateral (same)	Y^a^	Y	Y
IC467	52	Right	Y^a^	Y	Y
IC120	74	Right	Y	Y	
A4	74	Right	Y^a^		Y
A3	61	Bilateral (same)	Y^a^		Y
A2	63	Bilateral (same)	Y^a^		Y
A5	66	Right	Y^a^		Y
A6	75	Bilateral (same)	Y^a^		
A8	48	Bilateral (same)	Y		
A9	66	Left	Y^a^		
A10	54	Bilateral (same)	Y^a^		
A25	61	Right	Y^a^		
A11	72	Bilateral (same)	Y^a^		
A12	76	Bilateral (same)	Y^a^		
A61	68	Right	Y		
A29	57	Bilateral (same)	Y^a^		
A22	51	Bilateral (same)	Y^a^		
A13	50	Right	Y^a^		
A14	62	Bilateral (same)	Y^a^		
A15	45	Left	Y^a^		
A7	69	Right	Y^a^		
Adenofibroma					
IC450	27	Right (other)	Y^a^	Y	Y
Cystadenoma					
IC148	67	Bilateral (other)	Y^a^	Y	Y
IC24	77	Bilateral (same)	Y	Y	
IC196	46	unilateral (unspecified)	Y	Y	
IC7	79	Right	Y^a^	Y	
IC591	48	Right	Y	Y	
A17	35	Bilateral (same)	Y^a^		Y
A16	50	Right	Y^a^		
A18	73	Bilateral (same)	Y^a^		
A19	46	Right	Y^a^		
A20	65	Right	Y^a^		
A23	58	Bilateral (same)	Y^a^		
A21	64	Bilateral (same)	Y^a^		
A26	55	Bilateral (same)	Y^a^		
A27	59	Left	Y^a^		
A62	68	Bilateral (same)	Y		
A63	43	Right	Y		
A64	55	Bilateral (same)	Y		
A24	58	Bilateral (same)	Y^a^		
Normal					
IC79	60	n/a		Y	
IC236	n/a	n/a		Y	
IC369	40	n/a		Y	

Same = same diagnosis both ovaries. Other = different diagnosis in contralateral ovary. n/a, information not available. Y, included in this manuscript

^a^in Hunter et al. 2011

#### Copy number aberrations

Genome-wide copy number analysis was performed for eight unselected pure fibromas, and compared to copy number data from the stroma of 27 serous cystadenofibromas and 18 serous cystadenomas (collectively referred to as benign serous tumours). CNAs were detectable in 7/8 (88%) of the pure fibromas, with gain of chromosome 12 being the most recurrently observed aberration in five of eight (63%) cases (Additional file 1: Table S1). Other recurrent CNAs in the fibromas were gain of chromosomes 9 or 9q (50% cases), 18 and 21 (20% cases each). CNAs were detected in the stroma of 33% of benign serous tumours. Recurrent gain of chromosome 12 was also observed in 31% of serous cystadenofibromas (8/27) and 17% of serous cystadenomas (3/18), gain of 9q was only observed in single serous cystadenofibroma case, while loss of chromosome 22 was detected in 11% of cystadenofibromas (3/27). No CNAs were detected in the stroma of the normal ovaries.

#### Expression analysis

Gene expression arrays were used to compare the stromal RNA of three normal ovaries against eight pure fibromas (seven with CNAs), seven cystadenomas (two with CNAs), and seven cystadenofibromas (four with CNAs). Comparing normal ovary to benign serous tumours or fibromas did not identify any differentially expressed genes following multiple testing correction. Comparison of benign serous tumours to fibromas also did not identify any differentially expressed genes. Comparison of samples based on the presence or absence of CNA, or the presence of specific CNA compared to an absence of genomic aberrations (with and without tumour subtyping), did not identify differentially expressed genes that remained significant following multiple testing correction. As these samples are difficult to enrich for neoplastic cells due to a mixture of cell types in the stroma the expression signal from the tumour cells will be diluted, therefore a less stringent approach was taken to identify candidate genes by taking the most significantly altered genes p <=0.001 with a fold change >=2. Through this approach 17 genes were found to be differentially expressed based on the presence of specific CNA (gain 9q and 12, loss of 16q) compared to samples with no genomic CNAs (Table 2).

**Table 2:** Candidate genes for differential expression

Gene	Cytoband	Fold-change	*P* value	Function
9q genes gain vs no gain				
*HAPLN1*	5q14.3	+5.2	0.000008	ECM protein and ERK signalling; overexpressed in metastatic melanoma and mesotheliomas
*PRAME*	22q11.22	+3.6	0.000342	Repressor of retinoic acid receptor. Overexpressed in multiple neoplasms (including melanoma)
*SLC17A3*	6p22.2	+3.3	0.000081	Voltage-driven transporter. Affects serum uric acid levels
*CKS2*	9q22.2	+3.3	0.000232	CDC28 protein kinase regulatory subunit 2. Overexpressed in numerous neoplasms, overrides the intra-S-phase DNA damage checkpoint
*ANOS1*	Xp22.31	+2.8	0.000297	ECM protein. Putative cell adhesion molecule, upregulated in some tumour types
*RNF182*	6p23	+2.5	0.000110	E3 ubiquitin ligase. Overexpressed in Alzheimers
*SLC17A1*	6p22.2	+2.3	0.000711	Sodium-dependent phosphate transporter. Affects uric acid levels
*CRB1*	1q31.3	+2.1	0.000466	Photoreceptor protein
*SYT14*	1q32.2	+2.1	0.000601	Family of proteins involved in synaptic transmission
*C6orf115*	6q24.1	+2	0.000848	Uncharacterised protein
*APOD*	3q29	−2.6	0.000762	Putative lipoprotein metabolism. Inverse correlation between expression and colorectal tumour progression. Associated with neurodegeneration
*CLU*	8p21.1	−2.4	0.000751	Secreted anti-apoptotic chaperone protein. Overexpressed in many tumour types and associated with neurodegeneration
*SLFN11*	17q12	−2.3	0.000237	Putative DNA/RNA helicase. Expression of other family members inhibits growth of fibroblasts and thymoctyes. Sensitises cancer cells to DNA damaging agents
chr12 genes gain vs no gain				
*NDST3*	4q26	+2.9	0.000814	N-deacetylase/N-sulfotransferase 3. Golgi apparatus protein, associated with schizophrenia and bipolar disorder
*PRELP*	1q32.1	−2.2	0.000646	Connective tissue ECM protein. Abnormally expressed in chronic lymphocytic leukaemia cells
16q genes loss vs no loss				
*PRAME*	22q11.22	+6.4	0.000347	Repressor of retinoic acid receptor. Overexpressed in multiple neoplasms (including melanoma).
*GLRA2*	Xp22.2	+2.7	0.000412	Glycine receptor alpha 2, neutrophil and p38 MAPK associated
*GABRA5*	15q12	+2.5	0.000136	GABA receptor alpha 5, associated with schizophrenia and bipolar I disorder
*SLFN11*	17q12	−3.6	0.000336	Putative DNA/RNA helicase. Expression of other family members inhibits growth of fibroblasts and thymoctyes. Sensitises cancer cells to DNA damaging agents

#### Exome data

Exome sequencing was performed on the stromal DNA of seven cystadenofibromas, one adenofibroma and one cystadenoma (all with CNAs), and two cystadenofibromas and one cystadenoma with no CNAs. Exome sequencing identified 83 putative somatic variants, with an average of 7 mutations per case (range 2–20). It is difficult to enrich for the subpopulation of neoplastic fibroblasts in the stroma, as indicated by the low variant allele frequency of the majority of the variants (Additional file 2: Table S2), and subsequently difficult to validate findings by Sanger sequencing. We undertook Sanger validation of a subset of variants for each case. No recurrent mutations or recurrently mutated genes were identified that went on to validate. In total, 20 somatic variants were able to be validated by Sanger sequencing (Table 3). Of the validated variants, a single nonsense mutation was identified in the *DMD* gene. The remaining 19 validated variants were all missense variants, the functional impacts of which were assessed using transFIC (Transformed Functional Impact for Cancer) (Table 3). No variants from the three tumours with no CNAs validated by Sanger sequencing.

**Table 3:** Validated exome variants

Sample	CHR	POS	REF	ALT	Consequence	Gene	Amino acids	Condel	PolyPhen	SIFT	SiftTransficLabel	Validation
A42	11	821679	T	G	MS	*PNPLA2*	F/C	Deleterious (0.702)	Prob_damaging (0.984)	Tolerated (0.11)	Low_impact	Somatic
A42	19	897480	G	A	MS	*R3HDM4*	P/L	Deleterious (0.481)	Prob_damaging (0.996)	Tolerated (0.37)	Low_impact	Somatic
A4	2	197090514	T	C	MS	*HECW2*	Y/C	Deleterious (0.935)	Prob_damaging (0.999)	Deleterious (0)	High_impact	Somatic
A4	5	35068332	C	A	MS	*PRLR*	A/S	Neutral (0.019)	Benign (0.026)	Tolerated (0.38)	Medium_impact	Somatic
A4	16	9943623	C	T	MS	*GRIN2A*	V/I	Neutral (0.000)	Benign (0.002)	Tolerated (1)	Low_impact	Somatic
A4	19	4448308	C	G	SRV	*UBXN6*	.	.	.	.	.	Somatic
A4	19	38692604	G	C	MS	*SIPA1L3*	G/A	Neutral (0.001)	Benign (0.002)	Tolerated (0.82)	Low_impact	Somatic
A17	X	31222107	C	A	NS	*DMD*	E/*	.	.	.	.	Somatic
IC158	3	47127761	T	C	MS	*SETD2*	H/R	Deleterious (0.808)	Prob_damaging (0.96)	Deleterious (0.01)	Medium_impact	Somatic
IC158	1	44156598	C	T	MS	*KDM4A*	P/L	Deleterious (0.881)	Prob_damaging (0.987)	Deleterious (0)	High_impact	Somatic
IC158	7	96639132	C	T	MS	*DLX6*	R/C	Deleterious (0.877)	Prob_damaging (0.985)	Deleterious (0)	High_impact	Somatic
IC158	7	100484701	G	A	MS	*SRRT*	V/M	Deleterious 0.892)	Prob_damaging (0.992)	Deleterious (0)	Medium_impact	Somatic
IC158	14	102504858	G	T	MS	*DYNC1H1*	G/V	Deleterious (0.886)	Prob_damaging (0.99)	Deleterious (0)	High_impact	Somatic
IC158	16	21726339	G	A	MS	*OTOA*	A/T	Neutral (0.021)	Benign (0.042)	Tolerated (0.37)	Low_impact	Somatic
IC467	12	49422612	T	C	MS	*KMT2D*	K/R	Deleterious (0.543)	Prob_damaging (0.994)	Deleterious (0)	NA	Somatic
IC467	17	11603150	A	G	MS	*DNAH9*	K/E	Deleterious (0.832)	Prob_damaging (0.942)	Deleterious (0)	Medium_impact	Somatic
IC467	19	51582924	C	A	MS	*KLK14*	R/M	Neutral (0.346)	Benign (0.231)	Tolerated (0.06)	Medium_impact	Somatic
IC467	19	54313656	C	A	MS	*NLRP12*	E/D	Neutral (0.028)	Benign (0.113)	Tolerated (0.37)	Low_impact	Somatic
IC467	X	5811262	C	T	MS	*NLGN4X*	A/T	Neutral (0.199)	Poss_damaging (0.47)	Tolerated (0.21)	Low_impact	Somatic
IC467	X	77112855	T	C	MS	*MAGT1*	N/S	Neutra (0.002)	Benign (0.014)	Tolerated (0.76)	Low_impact	Somatic

*MS* missense, *NS* nonsense, *SRV* splice receptor variant

The ability to detect variants may be confounded by normal DNA contamination. There was a positive correlation between the number of variants detected (before validation) and the average allele frequency (Spearman’s r = 0.76, P = 0.01, Additional file 3: Figure S1). The mean sequencing depth was 121.3 reads (88.6–186.8) for germline samples and 122.7 reads (72.8–163.6) for somatic samples, therefore the detection of low frequency variants is not substantially compromised by limited depth of coverage.

### Discussion

The findings of this study are consistent with previous karyotyping and FISH studies that identified trisomy 12 as the most common chromosomal abnormality identifiable in ovarian fibromas [8–10]. Pure ovarian fibromas were found to harbour chromosomal abnormalities more frequently than benign serous ovarian tumours (88% vs. 33%), further supporting our hypothesis that a subset of benign serous tumours are actually fibromas that coincidentally have an associated epithelial cyst. However, benign serous tumours more frequently harboured trisomy 12 as the sole aberration (47% of tumours with aberrations) compared to fibromas (30% of tumours with aberrations). Fibromas also more frequently harboured CNA that were rarely detected in the benign serous tumours such as 9q gain (50% cases), potentially indicating unique underlying biological drivers.

Expression analysis provided some interesting candidates that have previously been associated with neoplasms or fibroblast growth for further investigation. Genes with increased expression in tumours with CNAs compared to those without CNAs included the extracellular matrix (ECM) and signalling molecules *HAPLN1* and *ANOS1*, the antigen and repressor of retinoic acid signalling molecule *PRAME*, and the cell cycle regulator *CKS2*. All of these genes have been previously associated with overexpression in other types of neoplasm [28–34], and *PRAME* and CKS2 expression have been proposed as markers of poor prognosis in high-grade serous ovarian carcinoma [33, 35].

Genes with decreased expression in tumours with CNAs compared to those without CNAs included the putative DNA/RNA helicase *SLFN11*, the ECM protein *PRELP*, the high density lipoprotein component *APOD*, and the secreted chaperone *CLU*. Although these have all been linked with altered expression in other neoplasms before, this has typically been upregulation and potentially linked to neoplastic progression [36–40], including in ovarian cancer for APOD and CLU [41, 42]. Data is inconsistent for *CLU*, as expression was also linked to improved prognosis in high-grade serous ovarian carcinoma [33]. Low expression of *SLFN11* has been associated with resistance to chemotherapy in ovarian cancer other cancers due to its role in the DNA damage response [43].

No genes were found to be recurrently mutated, however, two tumours had mutations in histone methytransferases (*SETD2* and *KMT2D)* and one also had a mutation in a demethylase (*KDM4A*), all previously associated with neoplasia and all mutations predicted to be deleterious. Other mutated genes have also been associated with neoplasia, such as *HECW2, SRRT* and *KLK14*, with predicted medium to high deleterious impact.

## Limitations

- Use of Affymetrix Human Gene 1.0 ST array is limited to the probes on the array at the time.
- Insufficient power to detect differentially expressed genes due to n = 3 normal ovaries.
- Limited ability to detect mutations due to normal contamination.

## Supplementary information

**Additional file 1: Table S1.** Genomic copy number aberrations.

**Additional file 2: Table S2**. Variants detected by exome sequencing.

**Additional file 3: Figure S1.** Correlation between the number of mutations and average allele frequency, suggesting that normal contamination may limit the number of mutations detected.

## Data Availability

Gene Expression Omnibus GSE67189-Molecular characterization of ovarian serous cystadenomas and fibromas [Copy number], GSE67223-Molecular characterization of ovarian serous cystadenomas and fibromas [Expression], GSE67224-Molecular characterization of ovarian serous cystadenomas and fibromas Sequence Read Archive PRJNA631561 (https://www.ncbi.nlm.nih.gov/sra/PRJNA631561).
