# Supplementary material for: Molecular comparison of pure ovarian fibroma with serous benign ovarian tumours
Source: BMC Res Notes. 2020 Jul 22;13:349. doi: 10.1186/s13104-020-05194-z (PMC7376903; doi:10.1186/s13104-020-05194-z)
Supplement: Supplementary file 1 — Additional file 1: Table S1. Genomic copy number aberrations. [file 13104_2020_5194_MOESM1_ESM.docx]

**Supplementary Table 1: Genomic copy number aberrations**

| **Sample ID** | **Histology** | **Stromal copy number aberrations** |
| --- | --- | --- |
| IC137^b^ | Fibroma | Gain: 12 |
| IC181^b^ | Fibroma | Gain: 12 |
| IC4^b^ | Fibroma | Gain: 4, 10, 12, 13, 17, 18p11.31-pter, 18p11.21-qter |
| IC269^b^ | Fibroma | Gain: 9, 12, 20 |
| IC33^b^ | Fibroma | Gain: 9q; LOH: 16q |
| IC494^b^ | Fibroma | Gain: 9q, 21 |
| IC458^b^ | Fibroma | Gain: 3, 5, 6, 7, 8, 9, 10, 12, 13, 14, 15, 18, 19, 20, 21, X (hypotriploid) |
| IC425^b^ | Fibroma | None |
| IC10^b^ | Serous cystadenofibroma | Gain: 12 |
| IC158^a,b,c^ | Serous cystadenofibroma | Gain: 12 |
| A2^a,c^ | Serous cystadenofibroma | Gain: 12 |
| A3^a,c^ | Serous cystadenofibroma | Gain: 12 |
| A5^a,c^ | Serous cystadenofibroma | Gain: 12 |
| A6 ^a^ | Serous cystadenofibroma | Gain: 12 |
| IC5^a,b,c^ | Serous cystadenofibroma | Gain: 12; LOH: 22 |
| A4^a,c^ | Serous cystadenofibroma | Gain: 12; LOH: 17q, 22 |
| IC450^a,b,c^ | Serous adenofibroma | LOH: 22 |
| IC467^a,b,c^ | Serous cystadenofibroma | Gain: 9q, 16q12.1-q12.2; LOH: 16q12.2-qter |
| A8 | Serous cystadenofibroma | None |
| A9 ^a^ | Serous cystadenofibroma | None |
| A10 ^a^ | Serous cystadenofibroma | None |
| IC103^a,b,c^ | Serous cystadenofibroma | None |
| IC120^b^ | Serous cystadenofibroma | None |
| IC149^b^ | Serous cystadenofibroma | None |
| IC164^a,b,c^ | Serous cystadenofibroma | None |
| A25 ^a^ | Serous cystadenofibroma | None |
| A61 | Serous cystadenofibroma | None |
| A11 ^a^ | Serous cystadenofibroma | None |
| A12 ^a^ | Serous cystadenofibroma | None |
| A29 ^a^ | Serous cystadenofibroma | None |
| A22^a^ | Serous cystadenofibroma | None |
| A13 ^a^ | Serous cystadenofibroma | None |
| A14 ^a^ | Serous cystadenofibroma | None |
| A15 ^a^ | Serous cystadenofibroma | None |
| A7 ^a^ | Serous cystadenofibroma | None |
| IC591^b^ | Serous cystadenoma | Gain: 12 |
| A62 | Serous cystadenoma | Gain: 10,12 |
| A17^a,c^ | Serous cystadenoma | Gain: 8, 10, 12, 13, 15, 18, 19 |
| A16 ^a^ | Serous cystadenoma | LOH: 3p21.33-14.3, 7q11.21-11.23, 7q22.1 |
| A63 | Serous cystadenoma | LOH: 3q13.31, 3q25.2-q26.33, 4q27,4q34.1, 4q35.1, 9p22.2, 14 |
| A64 | Serous cystadenoma | None |
| A18 ^a^ | Serous cystadenoma | None |
| A19 ^a^ | Serous cystadenoma | None |
| A20 ^a^ | Serous cystadenoma | None |
| A23 ^a^ | Serous cystadenoma | None |
| A21 ^a^ | Serous cystadenoma | None |
| A26 ^a^ | Serous cystadenoma | None |
| A27 ^a^ | Serous cystadenoma | None |
| A24 ^a^ | Serous cystadenoma | None |
| IC7^a,b^ | Serous cystadenoma | None |
| IC24^b^ | Serous cystadenoma | None |
| IC148 ^a,b,c^ | Serous cystadenoma | None |
| IC196^b^ | Serous cystadenoma | None |
| IC79^b^ | Normal ovary | None |
| IC236^b^ | Normal ovary | None |
| IC369^b^ | Normal ovary | None |

^a^Copy number data for these cases has been previously published (Hunter, 2011).

^b^Gene expression data also available for these samples (GSE67223)

^c^Exome data also available for these samples
