# Supplementary material for: Molecular comparison of pure ovarian fibroma with serous benign ovarian tumours
Source: BMC Res Notes. 2020 Jul 22;13:349. doi: 10.1186/s13104-020-05194-z (PMC7376903; doi:10.1186/s13104-020-05194-z)
Supplement: Supplementary file 3 — Additional file 3: Figure S1. Correlation between the number of mutations and average allele frequency, suggesting that normal contamination may limit the number of mutations detected. [file 13104_2020_5194_MOESM3_ESM.pdf]

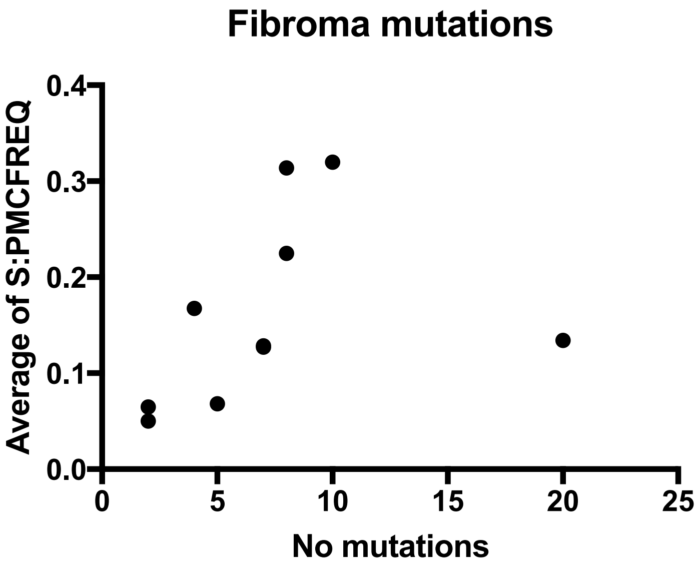

Supplementary Figure 1. Correlation between the number of mutations and average allele frequency, suggesting that normal contamination may limit the number of mutations detected.
